# Estimating the burden of rheumatoid arthritis in Africa: A systematic analysis

**DOI:** 10.7189/jogh.02.020406

**Published:** 2012-12

**Authors:** Ben Dowman, Ruth M. Campbell, Lina Zgaga, Davies Adeloye, Kit Yee Chan

**Affiliations:** 1 Centre for Population Health Sciences, The University of Edinburgh Medical School, Edinburgh, Scotland, UK; 2 Toronto General Hospital, University Health Network, Toronto, Canada; 3 Nossal Institute for Global Health, University of Melbourne, Melbourne, Australia; 4 Department of Health Policy and Management, School of Public Health, Peking University Health Science Centre, Beijing, China

## Abstract

**Background:**

Rheumatoid arthritis (RA) has an estimated worldwide prevalence of 1%. It is one of the leading causes of chronic morbidity in the developed world, but little is known about the disease burden in Africa. RA is often seen as a minor health problem and has been neglected in research and resource allocation throughout Africa despite potentially fatal systemic manifestations. This review aims to identify all relevant epidemiological literature pertaining to the occurrence of RA in Africa and calculate the prevalence and burden of disease.

**Methods:**

A systematic literature review of Medline, Embase and Global Health Library retrieved a total of 335 publications, of which 10 population studies and 11 hospital studies met pre–defined minimum criteria for relevance and quality. Data on prevalence was extracted, analysed and compared between population and hospital studies. Differences between genders were also analysed.

**Findings:**

The estimated crude prevalence of RA in Africa based on the available studies was 0.36% in 1990, which translates to a burden of 2.3 million affected individuals in 1990. Projections for the African population in 2010 based on the same prevalence rates would suggest a crude prevalence of 0.42% and the burden increased to 4.3 million. Only 2 population studies have been conducted after 1990, so projections for 2010 are uncertain. Hospital–based studies under–report the prevalence by about 6 times in comparison to population–based studies.

**Conclusion:**

The availability of epidemiological information on RA in Africa is very limited. More studies need to be conducted to estimate the true burden and patterns of RA before appropriate health policies can be developed.

Rheumatoid arthritis (RA) is one of the most common chronic diseases, with an estimated global prevalence of 1% [1], and is one of the leading causes of chronic morbidity in industrialised nations [2]. RA is an autoimmune disease that primarily affects the small joints of the hand, wrist, and feet. If left untreated, it can lead to extensive erosion of the cartilage, causing deformity and disability [3]. Common symptoms include pain and stiffness, but prolonged disease is also associated with psychological problems such as depression [3]. The cause of onset is currently unknown, but a genetic susceptibility to an environmental trigger seems the most plausible [4]. Various bacteria and viruses have been suggested as the initial trigger; with a form of molecular mimicry imitating human antigens activating an immune response against the host's own cells [3]. The disabilities caused by RA can have extensive impacts on quality of life, with loss of productivity due to damaged and deformed joints inhibiting fine movements of the hand [5]. This disability can lead to a loss of career and sources of income, which is a particular problem in low income settings. For a certain subset of the population, jobs in Africa involve a level of manual labour and the resource–starved African states can afford only limited or no welfare support for disabled individuals [2]. Along with the increase in non–communicable diseases (NCD) in developing countries, an increase in RA occurrence could stress medical services that are already struggling with a high burden of acute infectious illness to an extent that they may be unable to cope with the fast changing patterns of disease distribution seen in Africa today [6].

RA is not just a debilitating disease of small joints. It is also associated with significant extra–articular manifestations and mortality, with an average decreased life expectancy of 3–7 years in America [3]. Complications of RA can have extremely serious consequences, most commonly involving the respiratory, cardiac and visual systems [7]. The disease has been shown to increase the risk of stroke by up to 65% and through the development of rheumatic nodules the risk of myocardial infarctions, pleural effusions and lung infections is increased [7,8]. Since RA is an autoimmune disease, it can affect any part of the body, especially those that relay on small vessel beds or extensive nerve systems, and can contribute to the development of a whole plethora of life threating conditions [7]. The importance of NCDs in low and middle income countries has recently been internationally recognised by the United Nations (UN) as a problem that perpetuates and drives poverty and is a “threat to human, social, and economic development” [9,10]. Thereby, RA not only contributes significantly to this burden, but also contributes by increasing the rate of heart disease and stroke [7], certain cancers [11], and possibly diabetes [12]. RA is also a cause of gender inequality as it predominantly affects woman [3]. The prevention and management of RA could help reduce other NCDs by reducing shared risk factors and prevalence of systemic manifestations [13]. A historic perspective and specific considerations related to RA in the African context are provided in **Box 1** [2,4,5,14-22].

Box 1 **Rheumatoid arthritis in the African context**

The extent of the problem caused by RA in Africa is presently uncertain, as very few epidemiological studies have been conducted [14]. Historically, RA in Africa has been seen as a rare disease with a mild onset compared to Europe and America [15], and in the face of acute life threating illness such as HIV, malaria, tuberculosis, and severe malnutrition, it has been largely ignored and given a low priority for medical research and resource allocation [16]. RA has a significant impact on the burden of disease in many other developing regions of Asia and South America, so it is likely the situation is similar in Africa [2]. Most of the data for this low prevalence and mild course has come from studies conducted between the 1950s and 1980s [15,16]. Since the 1980s there has been a dearth of publications concerning RA, which is in opposition to the trends of disease research in Africa for other fields [17], and as such the current burden of RA remains unknown [2]. The limited data available appears to show an increasing prevalence and severity of RA in Africa, particularly for urban populations [15]. The literature would suggest that the first reported case in Africa has only been described in 1956 [18], since when an increasing number of cases have been identified across Africa. A good example is from a sequence of studies undertaken in a hospital in Mulago, Uganda, in which increasing number of patients with RA were identified over a period of decades. The first 6 confirmed cases were seen in 1969, but more than 400 further cases were seen in the same hospital by 1980 [15]. RA is believed to have first been described in the Americas, with evidence of its existence from records before European settlement; the first reported cases in Europe only date to the 17th century [4]. This would support the hypothesis of the disease being triggered by an interaction with an unknown infectious agent, and it would explain rare occurrence in rural populations, as any spread would initially be to large settlements with international connections [4,19]. In addition, the estimated prevalence of 0.2–0.3% in Africa is very small compared to the 2% seen in Jamaica [20].

Given that any reviews of the evidence will need to rely on an almost historic set of studies in Africa, one of the major concerns is how did political realities of the times in which the studies were conducted reflect upon the demographic structure of the study sample? Many studies in this review were conducted in South Africa, in the period between 1975 and 1988. In those times, it is possible that the studies were mainly addressing the prevalence of RA in the minority of European immigrant populations, while the studies conducted in other African countries may have also been disproportionately capturing the rates within the subpopulations of European colonizers. This is particularly likely to be true for hospital–based studies at the time.

Despite the scarcity of information regarding the prevalence of RA in Africa, some steps have been made to improve the coverage and support of the rheumatic diseases. In 1989 the African League of Associations for Rheumatology (AFLAR) was created in recognition of the large burden that this group of illness represents [21]. In association with the Community Oriented Program for Central Control of Rheumatic Diseases (COPCORD), created by WHO to record the burden caused by rheumatic disorders through populations surveys, AFLAR has been tasked to record and improve the care of rheumatic patients in Africa [22]. In diffidence to this initiative, only Egypt and Tunisia have a COPCORD centre in Africa, and only a single survey has been published regarding the prevalence of RA by AFLAR [22]. As it stands, very little significance is given to rheumatic disease in Africa, with only a single rheumatologist providing for the 16 million strong population of Kenya and thirty for the 40 million of South Africa as recently as 2003 [16].

Despite its serious health problems RA is a treatable disease if caught in the early stages [2]. A combination of disease–modifying anti–rheumatic drugs (DMARDs) and low dose steroids prescribed aggressively can drastically slow down the progression of the disease and even cause remission [2]. Unfortunately, the most effective biological drugs are very expensive and far beyond the means of resource–starved African countries [2]. An effective and cost-effective alternative to the front line drugs used in the industrial nations has been developed by COPCORD and can drastically alter the prognosis of the disease [5]. This set of treatment guidelines uses cheap generic drugs. Initially all 5 drugs are used in combination until remission is achieved. Once remission is seen, the intravenous drugs are withdrawn, leaving only oral drugs. The aim is to maintain remission without the need for any drug therapy as a stimulus [5]. This is a safe, cost-effective alternative to the usual high maintenance programs and highlights the need for epidemiological data to map the patterns of the disease, so that implementation can be started in the appropriate settings to maximise the cost-effectiveness of the programme. A degree of caution is warranted before certain DMARDs are prescribed in an Africa, as the immunosuppressant properties of the drugs can exacerbate problems associated with endemic infections such as TB, HIV, and malaria [16]. Some drug combinations have been shown to be safer than others but currently little research has been conducted and the safety parameters remain unknown [16].

The primary aim of this review will be to assess the prevalence of RA in Africa and to identify the key gaps in information. We will also study differences in RA prevalence between rural and urban populations, between male and female gender, and between hospital–based and community–based studies, aiming to identify all available, well conducted studies investigating the prevalence of RA in Africa.

## METHODS

### Search strategy

A systematic review of the public electronic databases, Ovid Medline (from 1946 onwards, in–process and non–indexed), Embase and Embase classic (from 1947), and Global Health Library (from 1910) was undertaken to find relevant literature. Searches using Google Scholar provided no additional references. Cross checking reference lists from review articles, editorials and study publications did not provide additional population based studies. The MeSH headings and key search terms used for Ovid Medline are described in **Table 1**. Search terms were altered when appropriate for other databases and the additional terms of prevalence, incidence and mortality were used alongside morbidity and burden of disease presented in the search strategy. All searches were completed on February 6th, 2012.

**Table 1:** Search terms used in the review of the literature

1.	(rheumatoid adj3 arthritis).af.
2.	exp arthritis, rheumatoid/ or exp caplan syndrome/ or exp felty’s syndrome/ or exp rheumatoid nodule/ or exp rheumatoid vasculitis/
3.	(rheum* adj3 arth*).tw.
4.	rheumatoid arthritis.tw.
5.	exp africa/ or exp africa, northern/ or exp algeria/ or exp egypt/ or exp libya/ or exp morocco/ or exp tunisia/ or exp “africa south of the sahara”/ or exp africa, central/ or exp cameroon/ or exp central african republic/ or exp chad/ or exp congo/ or exp “democratic republic of the congo”/ or exp equatorial guinea/ or exp gabon/ or exp africa, eastern/ or exp burundi/ or exp djibouti/ or exp eritrea/ or exp ethiopia/ or exp kenya/ or exp rwanda/ or exp somalia/ or exp sudan/ or exp tanzania/ or exp uganda/ or exp africa, southern/ or exp angola/ or exp botswana/ or exp lesotho/ or exp malawi/ or exp mozambique/ or exp namibia/ or exp south africa/ or exp swaziland/ or exp zambia/ or exp zimbabwe/ or exp africa, western/ or exp benin/ or exp burkina faso/ or exp cape verde/ or exp cote d'ivoire/ or exp gambia/ or exp ghana/ or exp guinea/ or exp guinea-bissau/ or exp liberia/ or exp mali/ or exp mauritania/ or exp niger/ or exp nigeria/ or exp senegal/ or exp sierra leone/ or exp togo/
6.	africa.tw.
7.	5 or 6
8.	1 or 2 or 3 or 4
9.	(burden and disease).tw.
10.	(disease adj3 burden*).tw.
11.	exp morbidity/
12.	morbidity.tw.
13.	9 or 10 or 11 or 12
14.	7 and 8 and 13

### Inclusion and exclusion criteria

As few studies were expected to have been published the initial inclusion criteria and search strategy was broad to minimise overlooking any publications. The inclusion criteria were: population or hospital based studies undertaken in Africa with a clearly expressed numerical form of disease prevalence showing a denominator population. A clear case definition was needed with the diagnosis of RA by a specified criteria of a modified or revised form of the Rome criteria [23], American Rheumatism Association (ARA) criteria of 1959, 1961 or 1968 [24-26], or the 1987 Criteria by the American College of Rheumatology (ACR) [27]. No date limit was applied.

Any non–population based studies were excluded from the main analysis, but hospital based studies were retained for the secondary analysis and review articles for a source of reference. Papers with incomplete detailing on the cohort population, papers that excluded prevalence data, papers based on unsound epidemiological methods and duplicated papers were all excluded. All forms of arthritis other than RA where excluded, as were forms of early onset RA (juvenile idiopathic arthritis), which is classified as a separate disease [28].

### Systematic analysis

**Figure 1:**
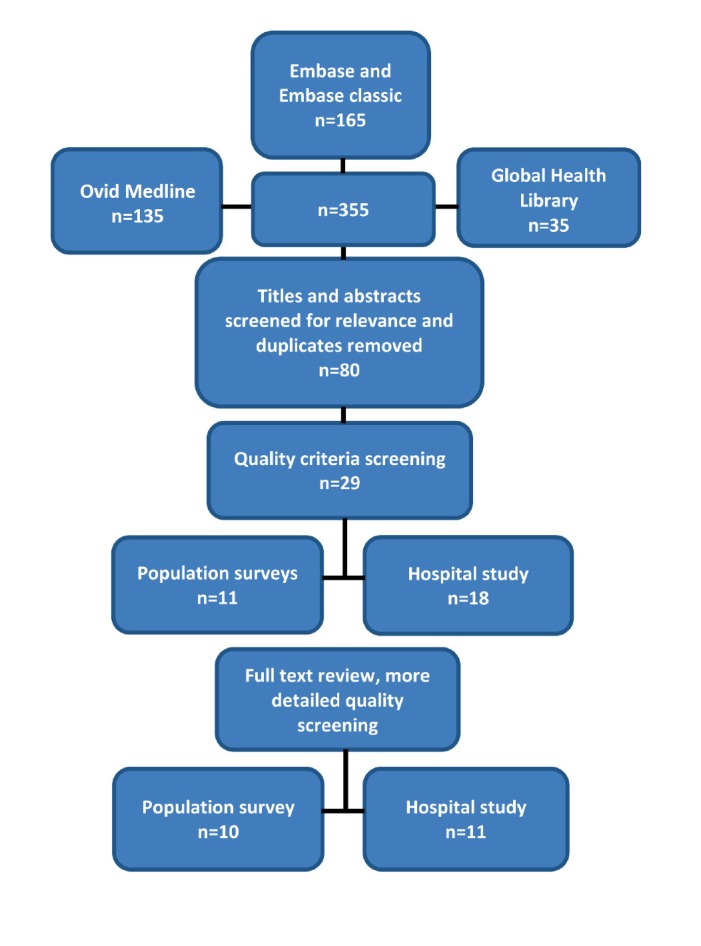
A summary of search strategy used in this study.

The initial search of Ovid Medline, Embase and Embase classic, and Global Health Library identified 335 papers. All papers deemed relevant after screening of titles were also screened using their abstracts; whenever abstracts were not available, full text sources were found and assessed. Any papers whose abstract or full text could not be located were appropriated through the InterLibrary Loan internet accessible database (ILLiad) and assessed once received. In total, 10 articles were received through ILLiad. Towards the end of the exclusion process, 80 articles were selected for abstract review, with 11 population studies and 18 hospital studies being identified after screening for quality and relevance. Reviews of full text articles retained only 10 population–based studies; 11 hospital studies were also kept for secondary aim of studying the difference in prevalence estimates between population and hospital–based studies. The literature search process is summarised in **Figure 1**.

### Data extraction and analysis

Selected studies where extracted and analysed in an Excel file. Information extracted included information on the studies such as the title, author(s), date, journal name, publication date and quantitative data. Specific quantitative information was recorded for the study size and number of reported RA cases calculated as a proportion. Wherever possible, information was broken into age groups for male, female, and combined populations. Cases of RA were defined as ‘probable’ or ‘definite’. The definition of ‘probable’ combined the American Rheumatism Association (ARA) and Rome criteria. The term ‘definite’ used the American College of Rheumatology (ACR) criteria. The setting of the paper, either rural or urban, was also recorded. This process was repeated for hospital–based studies.

The extracts of information typically contained the average age of a specific age group, the size of the population within this age group, and the reported prevalence. Whenever an open age bracket was given (ie, 75+), the average age for the age group was calculated using country population estimates from the closest year to the year of study using United Nations Population Division's (UNPD) data [29]. If the study date was between UNPD years the most recent year was used as the data was deemed more likely to be accurate. If no age break down was supplied, the data was combined into a single average age with prevalence and cohort size for the whole study. These data points were then combined into age subgroups of 10–year gaps, with the first interval having a 15 year gap (0–15, 15–25, 25–35, etc.). Different subgroups were used for hospital studies because of different data distribution. Once all the data was combined into a single table (see Online Supplementary Document(Online Supplementary Document)), a weighted prevalence was calculated using the cohort size for each data point. Once a prevalence and cohort size for each age subgroup has been found, 95% confidence intervals were calculated [30]. This process was repeated separately in male and female groups.

To assess the number of people suffering from RA in Africa, the weighted prevalence was calculated for each age group and applied to UNPD population data for Africa in 1990 [29]; 1990 was selected as it was the year to the period when most studies were conducted. The burden according to UNPD 2010 data was also calculated, using an estimated projection for the prevalence in 2010.

All processes specified for data analysis were repeated for hospital studies, population and hospital studies combined, rural studies, and urban studies. Confidence intervals were calculated for all of the aforementioned studies (see Online Supplementary Document(Online Supplementary Document)). Hospital–based studies were analysed separately from population–based studies because they are inherently biased and unrepresentative of the larger African population; only those individuals living in an urban environment, with enough money to pay for treatment, or in the end stages of severe illnesses, would be expected to seek medical attention [31]. Two of the identified studies had more than one distinct population group, so have been counted separately in the analysis. The study in the Western Cape of South Africa identified a rural cohort living on a reserve in Rietpoort, and an urban cohort living in Hout Bay [32]. Another study identified three cohorts, two of which were based in Nigeria and one in Liberia [33].

## RESULTS

### Study characteristics

**Figure 2:**
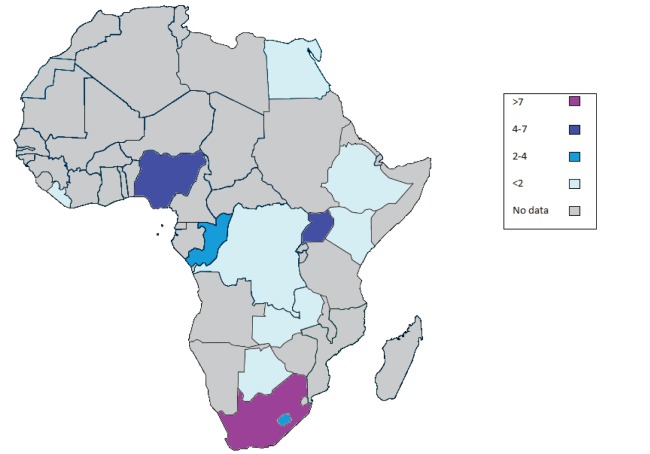
Geographic distribution of studies reporting prevalence of RA in Africa (number of studies from each country is indicated by different colours).

Only a small number of studies reporting the prevalence of RA were identified. The majority of studies were concentrated in a few countries (South Africa, Nigeria and Uganda), while for vast areas of Africa no data was available (**Figure 2**). In ten population–based studies the period of study, cohort size and setting varied widely. The date range was from 1968–2009, with the majority conducted in 1970s and 1980s. Only two papers were identified that were publishedafter 1990. The cohort sizes ranged from 35 to 5120 individuals. The majority of studies were conducted in rural settings. Three studies did not provide breakdown of their cohorts by age, and four (including those three) did not provide information on gender differences. Main study characteristics are provided in **Table 2**. Three different sets of criteria for defining RA were used: the Rome, ARA, and ACR criteria. A full list of hospital–based study characteristics are available in Online Supplementary Document(Online Supplementary Document). Only three hospital–based studies were published after 1990, and three were conducted in a rural setting.

**Table 2:** Characteristics of 10 identified population–based studies on rheumatoid arthritis

Author	Country	Setting	Publication year	RA criteria	Cohort size	Prevalence (%)		Prevalence (%, men only)	Prevalence (%, women only)
Abdel-Nasser et al [4]	Egypt	rural	2009	ACR 1987 [4]	5120		0.29	–	–
Beighton et al [34]	South Africa	rural	1975	Rome 1963 [34]	801		0.87	1.60	0.40
Brighton et al [35]	South Africa	rural	1988	Rome 1963 [35]	543		0.00	0.00	0.00
Meyers et al [36]	South Africa	rural	1977	Rome 1963 w/ ARA exclusions [36]	433		2.30	–	–
Meyers et al [32]	South Africa	rural	1982	ARA 1968 [32,36]	127		0.79	–	–
Meyers et al [32]	South Africa	urban	1982	ARA 1968 [32,36]	35		0.57	–*	–
Moolenburgh et al [37]	Lesotho	rural	1986	ARA 1959 [37]	1070		1.80	1.80	1.80
Muller et al [33]	Uganda and Liberia	rural	1972	ARA 1961 [33]	607		2.47	3.10	1.90
Silman et al [38]	Nigeria	rural	1993	ACR 1987 [38]	1994		0.00	0.00	0.00
Solomon et al [39]	South Africa	urban	1975	Rome 1963 [39,40]	551		3.27	2.60	3.70
Truswell et al [41]	Botswana	rural	1968	–	154		0.00	0.00	0.00

RA – rheumatoid arthritis, ARA – American Rheumatism Association

### Findings

**Figure 3:**
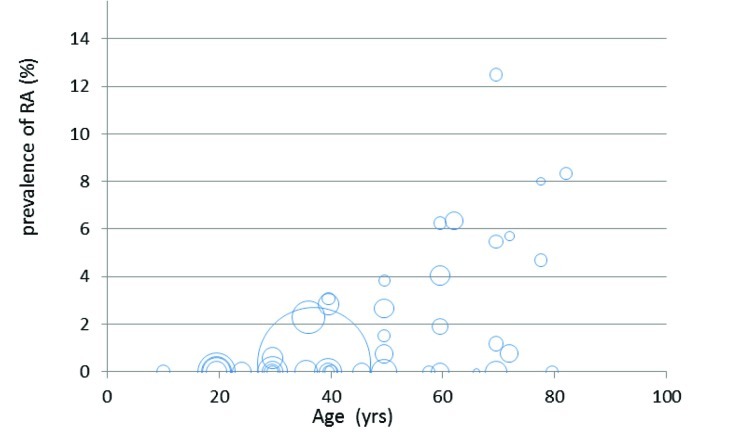
Relationship between the weighted mean age (in years) of examinees from 10 population–based studies belonging to different age groups and the observed prevalence (the size of bubbles is proportional to sample size of each sub–sample).

**Figure 4:**
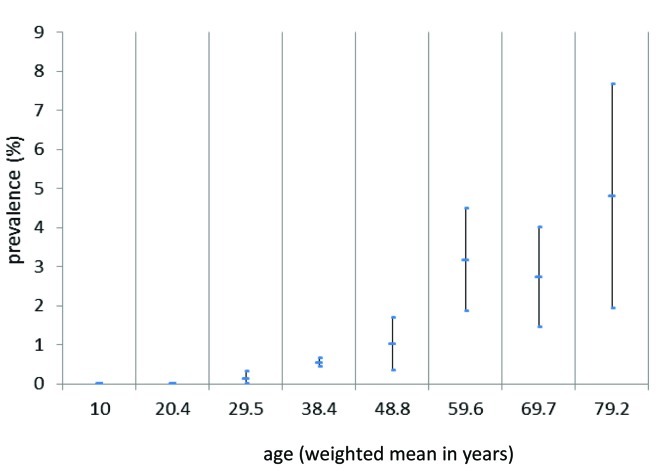
Relationship between the weighted mean age (in years) of examinees from 10 population–based studies belonging to different age groups and the observed prevalence (with 95% confidence intervals).

We extracted all the information on sub–samples from the 11 population–based studies which were defined by distinct age group, size of sub–sample in that age group, and reported prevalence for the age group. This database is visualized in **Figure 3**. There was an apparent increasing trend with age, which we further characterized by computing the weighted mean age for each group of sub–samples within the same age band, and assigning it a weighted mean prevalence and 95% confidence intervals (**Figure 4**). Using this information and the information on the demography of African population in 1990, we estimated that the crude prevalence of RA in Africa is 0.36%. This estimate is still considerably lower than the 1% for North America and Europe [4], but greater than the 0.2–0.3% which was a previous estimate for Africa [16]. If the prevalence in **Figure 4** was applied to the UNPD data for the African population size in 2010, crude prevalence would increase to 0.42%. These figures would translate into an overall burden of disease of over 2.5 million of people living with disease in Africa according to 1990 demographic profiles, but more than 4 million individuals in 2010 (see Online Supplementary Document(Online Supplementary Document) for rationales and methods behind these estimates).

Although the overall prevalence for Arica is 0.36%, there is a large variations in prevalence with age, sex, geographical region and setting. A large difference in the reported burden of RA was noted between population and hospital studies, with almost a 10-fold reduction in reporting from hospital reports (Online Supplementary Document(Online Supplementary Document)).

## DISCUSSION

Our literature review identified a number of population–based studies conducted in Africa that provided information on RA prevalence. To our knowledge, this study is the first systematic literature review that has combined all available publications on epidemiology of RA for Africa. Still, the scarcity of available information limits the generalizability of the results presented in this manuscript. The consistent trend of prevalence increasing with age was seen in every study. The difference in reported prevalence between population and hospital studies is substantial, with a 6:1 ratio in favour of population studies, with a likely explanation that fewer individuals with RA seek hospital care and most of them remain undiagnosed. Patients with acute illness such as infections or major trauma are more likely to seek medical attention, while hospitals themselves are unlikely to focus on identifying cases of RA given their burden and lack of resources [35,42]. An unexpected finding of this study was the similarity between the rates reported for males and females [35,38]. There are many possible explanations, including biases inherent to recruiting male individuals in rural population (with predominantly older and ill persons taking part), and cultural sensitivities surrounding the positive diagnosis in females [33].

Attempts were made to reduce bias as much as possible. A high sensitivity search criteria with abstract screening was implemented to identifying as many publications as possible. By extending the search criteria to the entire Africa from before and after 1990 a larger number of studies were identified. The inclusion of North African states is often questioned, as their ethnic and cultural background is distinct from Sub–Saharan Africa [43]. The inclusion of publications from before 1990 was a necessity as only two studies have been conducted after 1990s. Older studies have a different demographic pattern, generally being smaller and younger than contemporary populations [29]. This is why we used the population of Africa in 1990 as the most appropriate to estimate the overall burden.

Given that our review is based on an almost historic set of studies in Africa, one of the major concerns is how did political realities of the times in which the studies were conducted reflect upon the demographic structure of the study sample? Many studies in this review were conducted in South Africa, in the period between 1975 and 1988. In those times, it is possible that the studies were mainly addressing the prevalence of RA in the minority of European immigrant populations, while the studies conducted in other African countries may have also been disproportionately capturing the rates within the subpopulations of European colonizers. This is particularly likely to be true for hospital–based studies at the time.

One of the critical issues when estimating prevalence based on studies which span over such a long period of time is to ensure that diagnostic criteria did not change. Before 1990 two sets of criteria were used: the Rome and ARA. The ARA criteria are more clinically based and not always applicable to studying remote communities [35]. Even the simplified Rome criteria would be difficult to implement, due to the requirement of a detailed history of polyarthritis. Interpreters are often needed and detailed histories and can lead to ambiguous responses. Due to this difficulty, all papers using Rome definitions applied modified criteria that omitted past history, subsequently needing 2 of 3 positive findings from clinical, radiological and serological test being required to establish a diagnosis (rather than 3 of 4), which could lead to over–diagnosis. The ARA criteria appear to be more sensitive that the Rome set, but also prone to over–diagnosing [33]. Diagnostic tools, especially rheumatoid factor (RF) in serum, appear extremely unreliable [36,41,44]. Another problem in Africa is low participation rate and subsequent loss to follow up, due to simple superstitions and fears, cultural barriers and migration [45]. An incentive to motivate the population into attendance can greatly reduce rates of attrition, with Brighton et al 1988 having a 97% response rate due to a free health clinic set up in parallel with the study to support the local population [35].

A crude prevalence of 0.36% implied through these rare conducted studies, with a resulting burden of over 2 million persona in 1990 and more than 4 million in 2010, is relatively small compared to that in industrialized countries. However, it is still an important cause of morbidity. Given the limitations of the data presented here, any resource allocation based on these estimates would also need to be provisional. The most important policy implication from this review is an urgent need for more studies to be conducted. The publications used to generate an estimate is based on studies more than 20 years old and concentrated in a few countries, with the vast majority of Africa having no information over the past 50 years. A distinct correlation between age and prevalence is evident, but the usual gender ratios were not observed. More studies with better controlling for population bias are needed to confirm whether this is a true finding, so that programmes can be tailored to target high risk groups more effectively. This paper succeeded in revealing the importance of population–based estimates over hospital–based ones, as extensive under–reporting was noticed in hospital–based studies.

The basic infrastructure, with the African League of Associations for Rheumatology (AFLAR), is already present and should be built on to improve the coverage and research of RA. Despite the creation of AFLAR, little has been done to further the understanding of the epidemiology of RA in Africa. Cheap and effective treatments are available [5], so it is imperative that more studies are conducted to identify the true burden of RA and properly implement treatment.

Currently, this systematic review presents the only available systematic study of the burden of RA in Africa and it is suggestive of a larger prevalence than previously thought. This estimate is based on limited data, and further research is needed to consolidate our understanding of the true prevalence. Suggestions for future research are to use full (or randomly selected) area census, with a large cohort size (of more than 2000–3000 participants) using the ACR criteria as the most reliable diagnostic criteria currently available [27]. Local groups should be involved, particularly AFLAR and local clinics, as they have an existing capacity to monitor the population and their involvement may encourage increased interest and awareness of RA as an emerging African health problem.
