# Supplementary material for: Estimating the burden of rheumatoid arthritis in Africa: A systematic analysis
Source: J Glob Health. 2012 Dec;2(2):020406. doi: 10.7189/jogh.02.020406 (PMC3529310; doi:10.7189/jogh.02.020406)
Supplement: Online Supplementary Document [file jogh-02-020406-s001.pdf]

Online Supplementary Document

Dowman et al. Estimating the burden of rheumatoid arthritis in Africa: A systematic analysis

Journal of Global Health 2012;2:020406

Appendix 1: Data extracted from population-based studies
Appendix 2: Data extracted from hospital-based studies
Appendix 3: Data distribution by age-groups before weighting
Appendix 4: Graphs presenting data distribution
Appendix 5: Estimates of prevalence with 95% confidence intervals
Appendix 6: Burden of disease for African population using UNPD demographic data

Appendix 1: Data extracted from population-based studies

Truswell AS, Hansen JDL. (1968)				
Combined				
age distribution	mean age	cohort size	No. RA	prevalence
<20	10	72	0	0
20-60	40	68	0	0
60-72	66	15	0	0
total		154	0	0

Muller AS, Valkenburg HA, Greenwood BM. (1972)				
Combined. ARA				
age distribution	mean age	cohort size	No. RA	prevalence
25-34	29.5	173	1	0.58
35-44	39.5	176	5	2.84
45-54	49.5	134	1	0.75
55+	62	124	8	6.35
total		607	15	2.47
Male. ARA				
age distribution	mean age	cohort size	No. RA	prevalence
25-34	29.5	81	0	0
35-44	39.5	86	3	3.5
45-54	49.5	64	1	1.6
55+	62	64	5	7.8
total		295	9	3.1
Female. ARA				
age distribution	mean age	cohort size	No. RA	prevalence
25-34	29.5	92	1	1.1
35-44	39.5	90	2	2.2
45-54	49.5	70	0	0
55+	62	60	3	5
total		312	6	1.9

Beighton P, Solomon L, Vakenburg HA. (1975)				
Combined. Rome				
age distribution	mean age	cohort size	No. RA	prevalence
15-24	19.5	380	0	0
25-34	29.5	50	0	0
35-44	39.5	51	0	0
45-54	49.5	66	1	1.51
55-64	59.5	105	2	1.9
65-74	69.5	85	1	1.18
75+	77.5	64	3	4.69
total		801	7	0.87
Male. Rome				
age distribution	mean age	cohort size	No. RA	prevalence
15-24	19.5	182	0	0
25-34	29.5	17	0	0
35-44	39.5	18	0	0
45-54	49.5	20	1	5
55-64	59.5	31	2	6.5
65-74	69.5	24	1	4.2
75+	77.5	19	1	5.3
total		311	5	1.6
Female. Rome				
age distribution	mean age	cohort size	No. RA	prevalence
15-24	19.5	198	0	0
25-34	29.5	33	0	0
35-44	39.5	33	0	0
45-54	49.5	46	0	0
55-64	59.5	74	0	0
65-74	69.5	61	0	0
75+	77.5	45	2	4.4
total		490	2	0.4

Solomon L, Robin G, Valkenburg HA. (1975)				
Combined. Rome				
age distribution	mean age	cohort size	No. RA	prevalence
15-24	19.5	179	0	0
25-34	29.5	99	0	0
35-44	39.5	65	2	3.08
45-54	49.5	52	2	3.85
55-64	59.5	67	4	6.25
65-74	69.5	64	8	12.5
75+	77.5	25	2	8
total		551	18	3.3
Male. Rome				
age distribution	mean age	cohort size	No. RA	prevalence
15-24	19.5	70	0	0
25-34	29.5	34	0	0
35-44	39.5	25	2	8
45-54	49.5	16	1	6.3
55-64	59.5	23	0	0
65-74	69.5	21	2	9.5
75+	77.5	8	0	0
total		197	5	2.6
Female. Rome				
age distribution	mean age	cohort size	No. RA	prevalence
15-24	19.5	109	0	0
25-34	29.5	65	0	0
35-44	39.5	40	0	0
45-54	49.5	36	1	2.8
55-64	59.5	44	4	9.1
65-74	69.5	43	6	14
75+	77.5	17	2	11.8
total		354	13	3.7

Meyers OL, Daynes G, Beighton P. (1977)				
Combined. Rome w/ ARA exclusion criteria				
age distribution	mean age	cohort size	No. RA	prevalence
18+	36	433	10	2.3
total		433	10	2.3

Meyers OL, Jessop S, Klemp P. (1982)				
Combined. ARA				
age distribution	mean age	cohort size	No. RA	prevalence
65+	71.9	127	1	0.79
total		127	1	0.79

Meyers OL, Jessop S, Klemp P. (1982)				
Combined. ARA				
age distribution	mean age	cohort size	No. RA	prevalence
65+	71.9	35	2	5.71
total		35	2	5.71

Moolenburgh JD, Valkenburg HA, Fourie PB. (1986)				
Combined. ARA				
age distribution	mean age	cohort size	No. RA	prevalence
15-24	19.5	325	0	0
25-34	29.5	167	0	0
35-44	39.5	130	0	0
45-54	49.5	150	4	2.67
55-64	59.5	148	6	4.05
65-74	69.5	73	4	5.48
75+	82.1	60	2	8.33
total		1070	16	1.8
Male. ARA				
age distribution	mean age	cohort size	No. RA	prevalence
15-24	19.5	96	0	0
25-34	29.5	19	0	0
35-44	39.5	27	0	0
45-54	49.5	37	0	0
55-64	59.5	50	2	4
65-74	69.5	25	0	0
75+	82.6	23	0	0
total		280	2	1.8
Female. ARA				
age distribution	mean age	cohort size	No. RA	prevalence
15-24	19.5	229	0	0
25-34	29.5	150	0	0
35-44	39.5	103	0	0
45-54	49.5	113	4	3.5
55-64	59.5	98	4	4.1
65-74	69.5	58	4	6.9
75+	82.2	37	2	5.4
total		790	14	1.8

Brighton SW, de la Harpe AL, van Staden DJ, Badenhorst JH, Myers OL. (1988)				
Combined. Rome				
age distribution	mean age	cohort size	No. RA	prevalence
18-30	24	151	0	0
31-40	35.5	203	0	0
41-50	45.5	129	0	0
51+	57.5	60	0	0
total		543	0	0

Silman AJ, Ollier W, Holligan S, Birrell F, Adebajo A, Asuzu MC, Thomson W, Peeper L. (1994)				
Combined. ACR				
age distribution	mean age	cohort size	No. RA	prevalence
15-24	19.5	574	0	0
25-34	29.5	369	0	0
35-44	39.5	293	0	0
45-54	49.5	258	0	0
55-64	59.5	134	0	0
65-74	69.5	192	0	0
75+	79.5	60	0	0
total		1994	0	0
Male. ACR				
age distribution	mean age	cohort size	No. RA	prevalence
15-24	19.5	248	0	0
25-34	29.5	139	0	0
35-44	39.5	98	0	0
45-54	49.5	92	0	0
55-64	59.5	84	0	0
65-74	69.5	70	0	0
75+	79.1	23	0	0
total		757	0	0
Female. ACR				
age distribution	mean age	cohort size	No. RA	prevalence
15-24	19.5	326	0	0
25-34	29.5	230	0	0
35-44	39.5	195	0	0
45-54	49.5	166	0	0
55-64	59.5	150	0	0
65-74	69.5	122	0	0
75+	79.7	37	0	0
total		1237	0	0

Abdel-Nasser A, Abdel-Tawab R, Mahmoud J, Sammy A, Abdel-Fattah M. (2009)				
Combined. ACR				
age distribution	mean age	cohort size	No. RA	prevalence
15+	37	5120	18	0.35
total		5120	18	0.35
Male. ACR				
age distribution	mean age	cohort size	No. RA	prevalence
15+	33.6	2558	3	0.12
total		2558	3	0.12
Female. ACR				
age distribution	mean age	cohort size	No. RA	prevalence
15+	39	2562	15	0.47
total		2562	15	0.47

Appendix 2: Data extracted from hospital-based studies

Shaper AG, Shaper L (1958) - Urban				
combined				
age distribution	mean age	cohort size	No. RA	prevalence
30-44	37	2845	5	0.0018
total		2845	5	0.0018

Greenwood BM (1968) - Urban				
combined				
age distribution	mean age	cohort size	No. RA	prevalence
15-44	29.5	41798	32	0.08
45-64	54.5	11421	10	0.09
total		98454*	42	0.04

* Total population includes age groups outside age distribution. Not broken down in separate age groups in study

Greenwood BM (1969) - Urban				
combined				
age distribution	mean age	cohort size	No. RA	prevalence
	34.2	67377	35	0.05
total		67377	35	0.05

Anderson IF (1970) - Urban				
combined				
age distribution	mean age	cohort size	No. RA	prevalence
21-30	25.5	10503	6	0.06
31-40	35.5	7827	7	0.09
41-50	45.5	5753	3	0.05
51-60	55.5	4014	3	0.07
61+	68.9	3680	4	0.11
total		66900*	23	0.03

* Total population includes age groups outside age distribution. Not broken down in separate age groups in study

Edgington ME, Hodkinson J, Seftel HC (1972) - Rural combined				
age distribution	mean age	cohort size	No. RA	prevalence
10-20	15.00	81	0	0
21-30	25.50	124	1	0.81
31-40	35.5	129	0	0
41-50	45.5	102	2	1.96
51-60	55.5	54	0	0
61+	68.9	43	0	0
total		533	3	0.56

Tsega E, Choremi H, Bottazzo GF, Doniach D (1980) - Urban				
combined				
age distribution	mean age	cohort size	No. RA	prevalence
†	21.9	7966	19	0.24
total		7966	19	0.24

† Age groups not given. Estimation of mean age from UNPD data

Moolenburgh JD, Moore S, Valkenburg HA, Rrasmus MG. (1984) - Rural				
combined				
age distribution	mean age	cohort size	No. RA	prevalence
15-24	19.5	5046	2	0.04
25-34	29.5	4698	8	0.17
35-44	39.5	2262	5	0.22
45-54	49.5	1566	8	0.51
55-64	59.5	1218	5	0.41
65+	72.8	1044	4	0.38
total		15834	32	0.2

Brighton SW, de la Harpe AL, van Staden DJ, Badenhorst JH, Myers OL (1988) - Rural				
combined				
age distribution	mean age	cohort size	No. RA	Prevalence
*	22.3	255740	14	0.0055
total		255740	14	0.0055

* Age groups not given. Estimation of mean age from UNPD data

Bwanahali K, Mbuyi M, Kapita B (1991) - Urban				
combined				
age distribution	mean age	cohort size	No. RA	Prevalence
11-20	15.5	420	1	0.24
21-30	25.5	791	4	0.51
31-40	35.5	664	4	0.6
41-50	45.5	459	1	0.22
51-60	55.5	230	1	0.43
61-70	65.5	107	0	0
71+	75.6	30	0	0
total		2701	11	0.41
male				
age distribution	mean age	cohort size	No. RA	Prevalence
11-20	15.50	152	0	0
21-30	25.5	419	1	0.2
31-40	35.5	305	2	0.66
41-50	45.5	221	0	0
51-60	55.50	133	0	0
61-70	65.5	52	0	0
71+	72.5	13	0	0
total		1295	3	0.23
female				
age distribution	mean age	cohort size	No. RA	Prevalence
11-20	15.5	268	1	0.37
21-30	25.5	372	3	0.81
31-40	35.5	359	2	0.84
41-50	45.5	238	1	1.03
51-60	55.5	97	1	1.82
61-70	65.5	55	0	0
71+	75.7	17	0	0
total		1406	8	0.57

Bileckot R, Malonga AC (1998) - Urban				
combined				
age distribution	mean age	cohort size	No. RA	Prevalence
23-65	43.5	3518	36	1.02
total		3518	36	1.02

Malemba JJ, Mbuyi-Muamba JM (2007) - Urban				
combined				
age distribution	mean age	cohort size	No. RA	Prevalence
*	45.3	19587	82	0.49
total		19587	82	0.49
male				
age distribution	mean age	cohort size	No. RA	Prevalence
*	44.3	9753	22	0.23
total		9753	22	0.23
FEMALE				
age distribution	mean age	cohort size	No. RA	Prevalence
*	46.5	9852	60	0.61
total		9852	60	0.61

* Age groups not given. Estimation of mean age from UNPD data

Appendix 3: Data distribution by age -groups before weighting

<u>Population studies: both sexes</u>

AGE	Prevalence	Cohort size
10	0	72
**19.5**	**0**	**380***
**19.5**	**0**	**179**
**19.5**	**0**	**325**
**19.5**	**0**	**574**
**24**	**0**	**151**
29.5	0	167
29.5	0	99
29.5	0	50
29.5	0.58	173
29.5	0	369
**35.5**	**0**	**203**
**36**	**2.3**	**433**
**37**	**0.35**	**5120**
**39.5**	**2.84**	**176**
**39.5**	**0**	**51**
**39.5**	**3.08**	**65**
**39.5**	**0**	**130**
**39.5**	**0**	**293**
**40**	**0**	**68**
45.5	0	129
49.5	0.75	134
49.5	1.51	66
49.5	3.85	52
49.5	2.67	150
49.5	0	258
**57.5**	**0**	**60**
**59.5**	**4.05**	**148**
**59.5**	**6.25**	**67**
**59.5**	**1.9**	**105**
**59.5**	**0**	**134**
**62**	**6.35**	**126**
66	0	15
69.5	1.18	85
69.5	12.5	64
69.5	5.48	73
69.5	0	192
71.9	0.79	127

71.9	5.71	35
**77.5**	**8**	**25**
**77.5**	**4.69**	**64**
**79.5**	**0**	**60**
**82.1**	**8.33**	**60**

Population studies: females only

AGE	Prevalence	Cohort size
19.5	0	182
19.5	0	70
19.5	0	96
19.5	0	248
**29.5**	**0**	**139**
**29.5**	**0**	**19**
**29.5**	**0**	**34**
**29.5**	**0**	**17**
**29.5**	**0**	**81**
**33.6**	**0.12**	**2558**
39.5	0	27
39.5	3.5	86
39.5	0	18
39.5	8	25
39.5	0	98
**49.5**	**0**	**92**
**49.5**	**6.3**	**16**
**49.5**	**5**	**20**
**49.5**	**1.6**	**64**
**49.5**	**0**	**37**
59.5	4	50
59.5	6.5	31
59.5	0	23
59.5	0	84
62	7.8	64
**69.5**	**4.2**	**24**
**69.5**	**9.5**	**21**
**69.5**	**0**	**25**
**69.5**	**0**	**70**
77.5	0	8
77.5	5.3	19
79.1	0	23
82.6	13	23

Population studies: males only

AGE	Prevalence	Cohort size
19.5	0	198
19.5	0	109
19.5	0	229
19.5	0	326
**29.5**	**0**	**230**
**29.5**	**0**	**150**
**29.5**	**0**	**65**
**29.5**	**0**	**33**
**29.5**	**1.1**	**92**
39	0.47	2562
39.5	2.2	90
39.5	0	33
39.5	0	40
39.5	0	103
39.5	0	195
**49.5**	**0**	**166**
**49.5**	**3.5**	**113**
**49.5**	**2.8**	**36**
**49.5**	**0**	**46**
**49.5**	**0**	**70**
59.5	0	74
59.5	9.1	44
59.5	4.1	98
59.5	0	150
62	5	60
**69.5**	**0**	**122**
**69.5**	**0**	**61**
**69.5**	**14**	**43**
**69.5**	**6.9**	**58**
77.5	11.8	17
77.5	4.4	45
79.7	0	37
82.2	5.4	37

Hospital studies: both sexes

AGE	Prevalence	Cohort size
15.5	0.24	420
19.5	0	179
**21.9**	**0.24**	**7966**
**25.5**	**0.06**	**10503**
**25.5**	**0.51**	**791**
**29.5**	**0**	**99**
**29.5**	**0.08%**	**41798**
34.2	0.05	67377
35.5	0.6	664
35.5	0.09	7827
37	0.0018	2845
39.5	3.08	65
**43.5**	**1.02**	**3518**
**45.3**	**0.49**	**19587**
**45.5**	**0.05**	**5753**
**45.5**	**0.22**	**459**
**49.5**	**3.85**	**52**
54.5	0.09	11421
55.5	0.43	230
55.5	0.07	4014
59.5	6.25	67
**65.5**	**0**	**107**
**68.9**	**0.11**	**3680**
**69.5**	**12.5**	**64**
71.9	5.71	35
75.6	0	30
77.5	8	25

Appendix 4: Graphs presenting data distribution

Population studies:

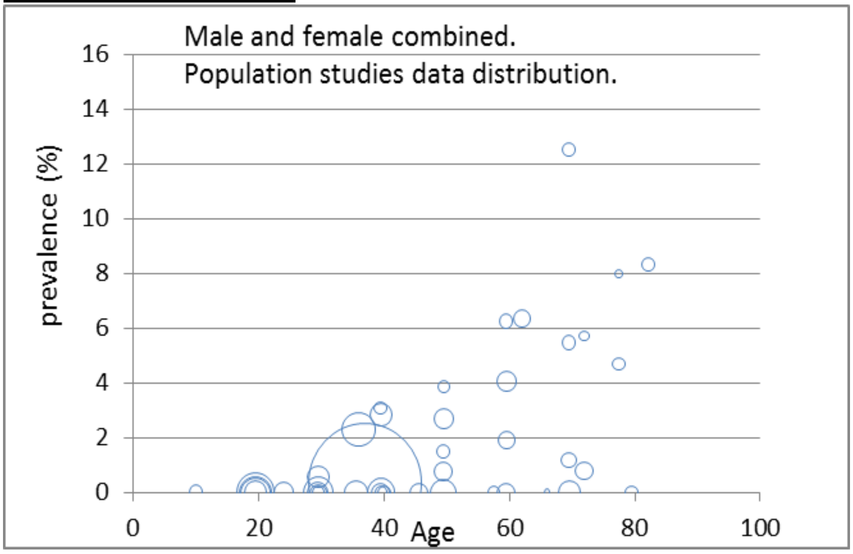

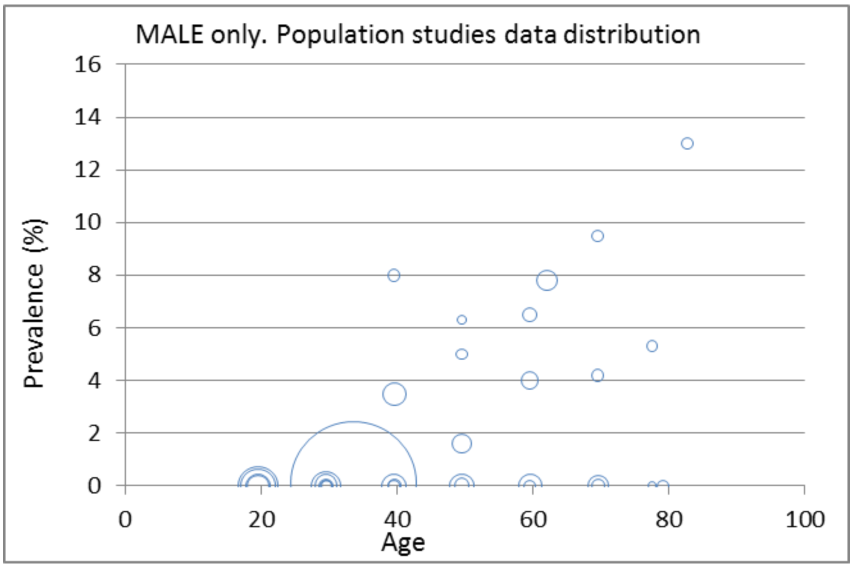

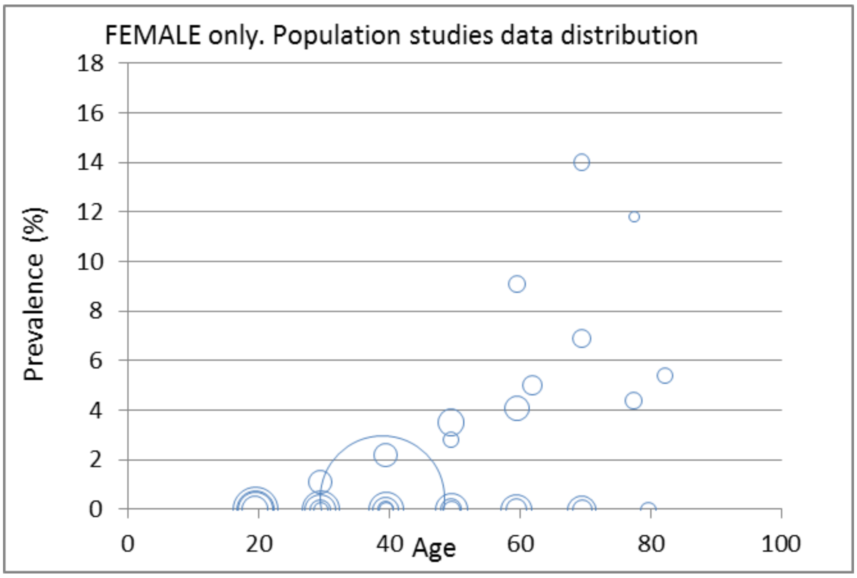

Hospital studies:

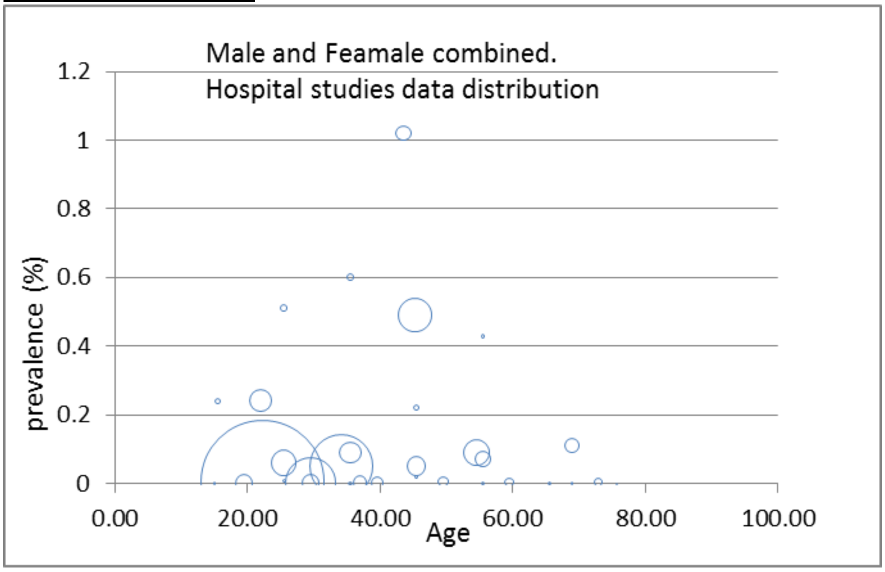

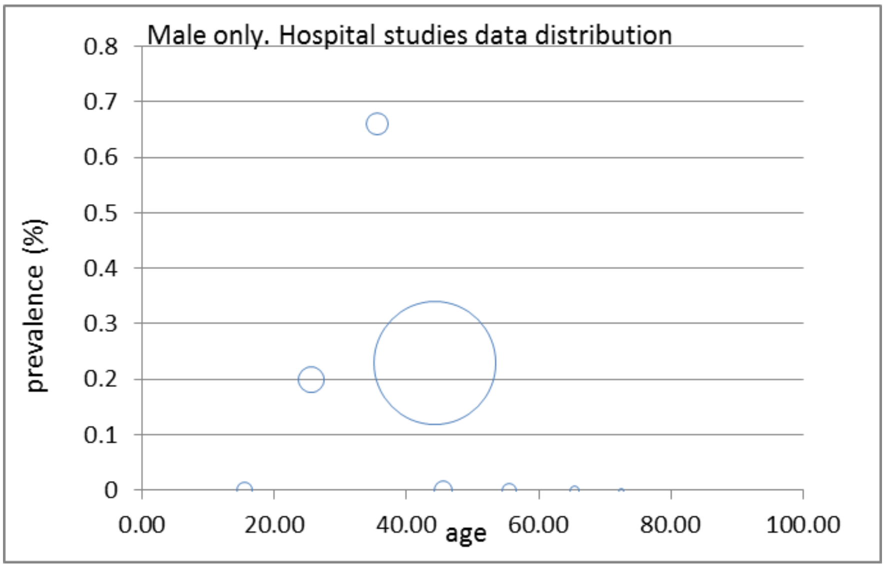

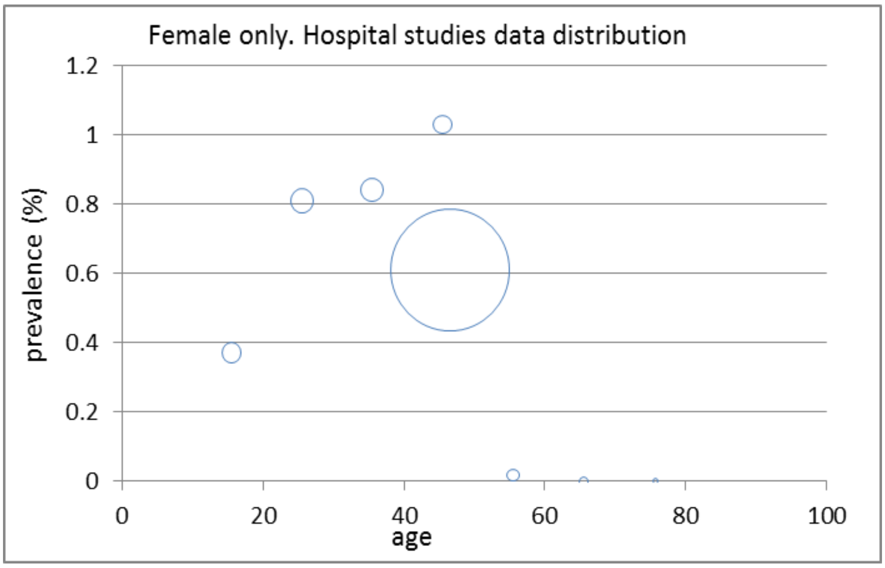

Appendix 5: Estimates of prevalence with 95% confidence intervals*

Population-based studies: both sexes

Age (years)	Prevalence (%)	cohort size	Confidence intervals 95%	
0-15	0	72	0	0
15-25	0	1609	0	0
25-35	0.117	858	0	0.305
35-45	0.533	6539	0.418	0.648
45-55	1.015	789	0.340	1.690
55-65	3.152	640	1.837	4.467
65-75	2.708	591	1.434	3.982
75+	4.784	209	1.917	7.651

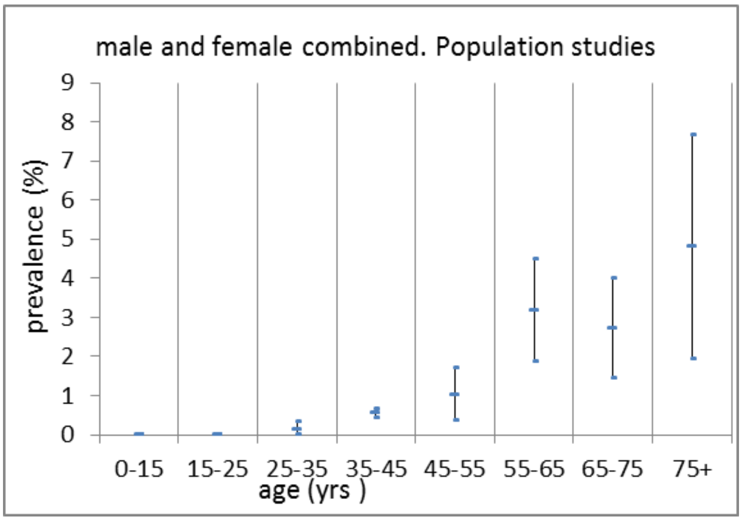

<u>Population-based studies: males only</u>

Age (years)	Prevalence (%)	cohort size	Confidence intervals 95%	
15-25	0	596	0	0
25-35	0.11	2848	0.04	0.18
35-45	1.97	254	0.31	3.63
45-55	1.32	229	0	2.76
55-65	3.57	252	1.35	5.79
65-75	2.15	140	0	4.51
75+	5.48	73	0.30	10.66

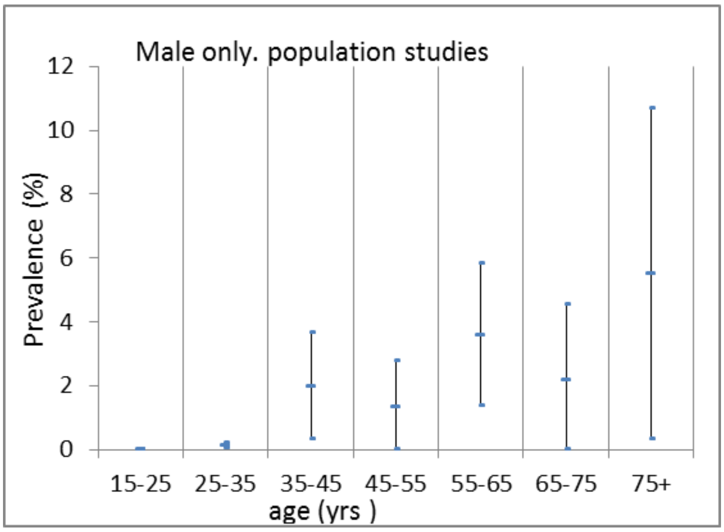

Age (years)	Prevalence (%)	cohort size	Confidence intervals 95%	
15-25	0	862	0	0
25-35	0.18	570	0	0.51
35-45	0.46	3023	0.35	0.63
45-55	1.15	431	0.18	2.12
55-65	2.59	426	1.14	4.04
65-75	4.04	284	1.81	6.27
75+	4.4	136	0.99	7.81

<u>Population-based studies: females only</u>

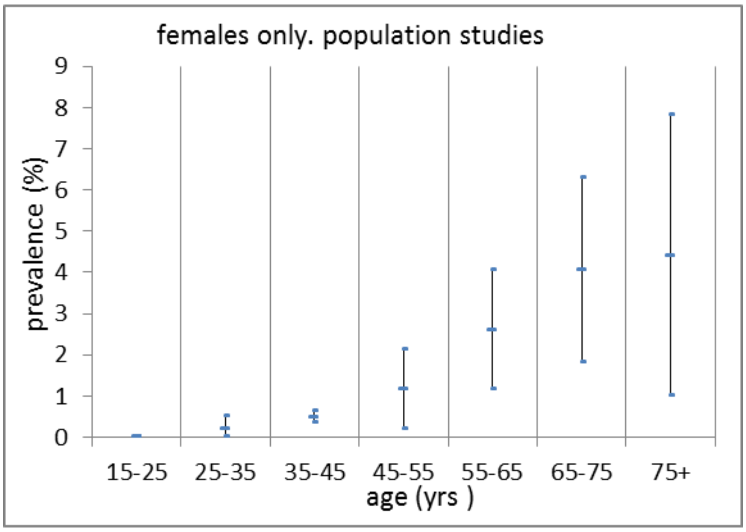

Hospital-based studies: both sexes

Age (years)	Prevalence (%)	cohort size	Confidence intervals 95%	
15-25	0	862	0	0
25-35	0.18	570	0	0.51
35-45	0.46	3023	0.35	0.63
45-55	1.15	431	0.18	2.12
55-65	2.59	426	1.14	4.04
65-75	4.04	284	1.81	6.27
75+	4.4	136	0.99	7.81

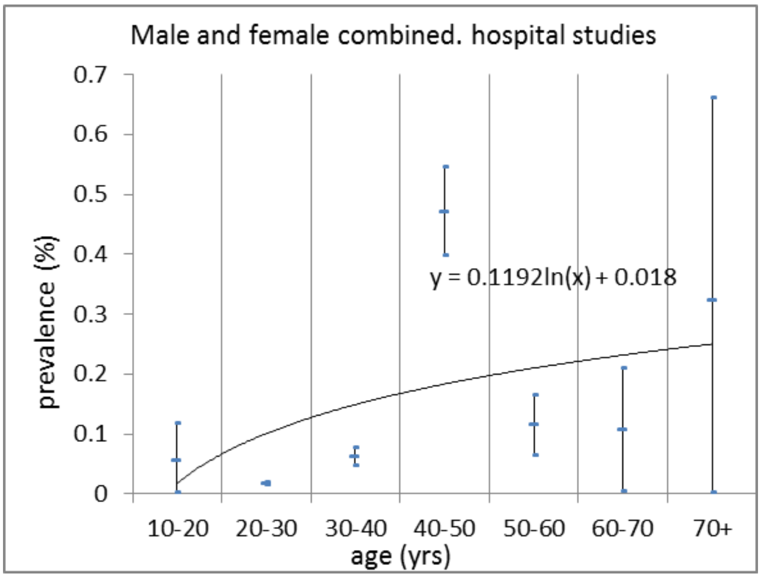

Appendix 6: Burden of disease for African population using UNPD demographic data

Burden of RA in the year 1990 - both sexes

age	burden of diseases	population of age group UNPD 1990
0-14	0	282,352,000
15-24	0	122,399,000
25-34	99,892	85,378,000
35-44	312,155	58,603,000
45-54	404,853	39,887,000
55-64	847,320	26,882,000
65-74	389,518	14,384,000
75+	258,383	5,401,000
total	2,311,124	635,287,000

Burden of RA in the year 2010 - both sexes

Age (years)	Burden of disease to the nearest 1000	Prevalence (%)	Population by UNPD 2010
0-14	0	0	411,728,000
15-24	0	0	205,355,000
25-34	179,000	0.117	153,166,000
35-44	537,000	0.533	100,750,000
45-54	705,000	1.015	69,492,000
55-64	1,443,000	3.152	45,793,000
65-74	683,000	2.708	25,213,000
75+	725,000	4.784	15,162,000
total	4,272,000	0.416	1,026,639,000
